# Simultaneous resection of abdominal aortic aneurysm and left renal cell carcinoma: A rare case and review of the literature

**DOI:** 10.34172/jcvtr.2020.26

**Published:** 2020-01-07

**Authors:** Mohammad Mozafar, Sina Zarrintan, R. Shane Tubbs

**Affiliations:** ^1^Division of Vascular & Endovascular Surgery, Department of General & Vascular Surgery, Shohada-Tajrish Hospital, Shahid Beheshti University of Medical Sciences, Tehran, Iran; ^2^Department of Neurosurgery, Tulane University School of Medicine, New Orleans, LA, USA; ^3^Department of Structural & Cellular Biology, Tulane University School of Medicine, New Orleans, LA, USA; ^4^Department of Anatomical Sciences, St. George’s University, Grenada

**Keywords:** Abdominal Aortic Aneurysm, Renal Cell Carcinoma, Nephrectomy

## Abstract

We report a rare case of concomitant abdominal aortic aneurysm (AAA) and left renal cell carcinoma (RCC). The patient was an 81-year old man who presented with vague abdominal pain. The investigations revealed a 110*73*62 mm AAA together with 69*56 left renal mass. Open repair of AAA with left radical nephrectomy was conducted. A simultaneous procedure is safe and does not increase morbidity and mortality in selected cases.

## Introduction

Concomitant renal cell carcinoma (RCC) and abdominal aortic aneurysm (AAA) is a rare entity.^1^ The association between AAA and RCC raises several questions and options for surgical management of this synchronous condition are of debate and potential clinical concern.^2^ Timing and type of procedures are decided based on patients’ condition.^3^ In the present report, we discuss the simultaneous open repair of AAA and radical nephrectomy.

## Case Report

*Initial presentation.* An 81-year old male patient referred to our vascular surgery clinic with a history of vague abdominal pain beginning from four months ago. The patient had diminished appetite, mild weight loss (5 kg) and generalized weakness. The patient did not have nausea, vomiting and change in bowel habits for the past four months. Anemia was present. There was not history of melena, hematemesis or hematochezia. The patient had history of hypertension and ischemic heart disease. He had also history of smoking (1 pack year) and opium use. The drug history was Nitrocontin 2.6 mg bid, losartan 25 mg daily, ASA 80 mg daily, atenolol 50 mg daily and pantoprazole 20 mg daily. The patient had a normal physical examination except for a pulsatile mass around his umbilicus. Upper and lower extremity pulses were normal.

**Figure 1:**
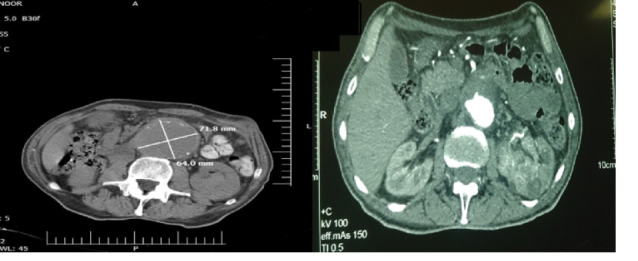
Computed tomography scan of the patient illustrating a 110*73*62 infra-renal abdominal aortic aneurysm together with a 69*65 mm mass lesion in left kidney.

*Lab and imaging findings.* The patient had a WBC count of 6000 per microliter. Hemoglobin concentration was 6.9 g/dL. Platelets were 430 000 per microliter. Electrolytes, serum creatinine, blood urea nitrogen, coagulation tests, liver functions tests and urine analysis were normal. Erythrocyte sedimentation rate (ESR) was 50 mm/h. An upper endoscopy and barium enema was conducted because of the persisting anemia. Both tests were normal. A computed tomography (CT) angiography of abdomen and pelvis was done. There was a 110*73*62 mm fusiform aneurysm in infra-renal abdominal aorta accompanied by mural thrombosis. Both common iliac arteries and their distal branches were normal (Figure 1; left). In addition, a 69*56 mm mass lesion was seen in the hilum of the left kidney. The mass had invasion to left renal vein (Figure 1; right).

**Figure 2:**
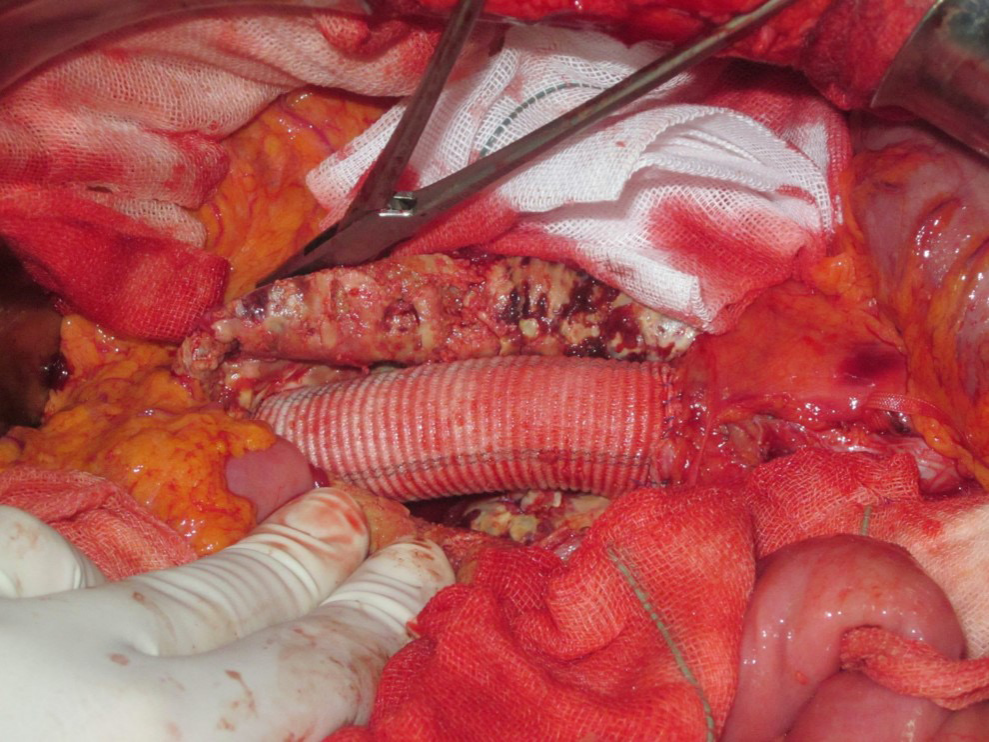
Open repair of abdominal aortic aneurysm by a tubular Dacron graft.

**Figure 3:**
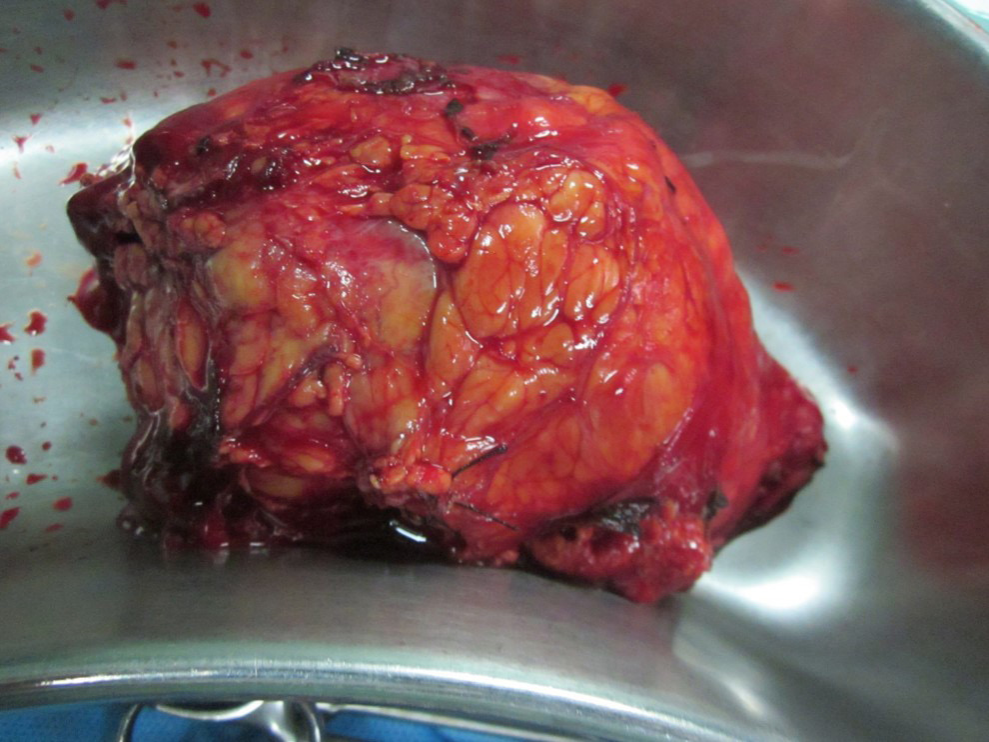
Left radical nephrectomy demonstrating a hilar mass lesion.

*Surgical management.* A single stage aneurysmectomy and left radical nephrectomy was planned. A midline laparotomy was conducted for transperitoneal repair of AAA. A tubular 22 mm Dacron graft was used to replace the infra-renal segment of the aorta (Figure 2). Following aneurysm repair, exploration of left kidney confirmed the presence of a 7*7 cm mass at the hilum. A left radical nephrectomy was performed (Figure 3). The patient tolerated the operation well and postoperative period was without any morbidity. The pathological examination of left kidney revealed clear cell type RCC.

## Discussion

Presentation of concomitant AAA and RCC is of potential concern and clinical interest.^4^ AAA is typically increased with aging and incidence of a number of malignancies is also increased by age. Thus, association of AAA and visceral malignancies is becoming more frequent.^2,5^ The therapeutic and surgical strategies of management of AAA and RCC are controversial regarding one-stage and two-stage approaches and also the priority of each entity.^1^ Nephrectomy is done by open or laparoscopic approach. AAA is approached either by open aneurysmectomy and graft replacement or by endovascular aneurysm repair (EVAR) technique.^5-7^ One-stage or simultaneous approach manages AAA (open repair or EVAR) and renal carcinoma in one operative session. However, in two-staged approach the aneurysm or renal carcinoma in managed first based on the clinical significance of each condition.^8-10^

In the present article, we report a case of an 81-old male with synchronous AAA and left RCC. We conducted simultaneous open AAA repair and open radical nephrectomy. Likewise, in most of the previously reported cases, simultaneous open nephrectomy and AAA surgery have been suggested as a standard and safe treatment.^2,6^ However, in high risk and elderly patients, the procedure could be life threatening and endovascular techniques would decrease morbidity and mortality. Currently, EVAR is the primary treatment method for the repair of infra-renal AAA due to improved short-term outcomes.^11^ In addition, moderate-quality evidence suggests that there is no difference in 30-day mortality between emergency EVAR and open repair.^12^ Thus, recent reports recommend EVAR technique followed by laparoscopic nephrectomy in two stages to manage synchronous AAA and RCC. Nephrectomy is also conducted laparoscopically.^5,7^

A comprehensive literature review revealed 94 cases of previously reported concomitant AAA and RCC cases. Table 1 demonstrates the studies reporting these cases. Of 94 reported cases, 73 patients (77.7%) had one-stage AAA and RCC management while 21 patients (22.3%) had two-stage operation. EAVR was used in 6 patients (6.4%)^3,5,7,15^ and open AAA repair was conducted in 88 patients (93.6%). Open nephrectomy was conducted in 90 patients (95.7%). Laparoscopic nephrectomy was used in two patients (2.1%).5,7 In one patient (1.1%), the accessory lower pole renal artery which supplied the tumor was ligated and tumor regression was evident.^15^ Two patients had horseshoe kidney, RCC and AAA. In one patient, a left radical nephrectomy with the division of the isthmus was conducted and AAA was reconstructed with an artificial graft.^13^ The other case had an AAA with a horseshoe kidney and an isthmus mass. Endovascular management of AAA was conducted and two accessory renal arteries believed to feed the isthmus mass were sacrificed.^15^ Horseshoe kidney regardless of RCC could also be associated with AAA.^19,20^ Preoperative arteriography and venography has been proposed to clarify the anatomy preoperatively.^13^ Endovascular techniques are safe and feasible in the management of synchronous AAA and horseshoe kidney even in the presence of malignancy.^15^

**Table 1:** Literature review presenting previously reported cases of concomitant abdominal aortic aneurysm and renal cell carcinoma

**Author(s)**	**Year**	**Patient(s)**	**Procedure**	**AAA**	Nephrectomy*
Baskin et al^8^	1991	1	One-stage	Open	Open nephrectomy
DeMasi et al^6^	1994	5	One-stage:4 Two-stage:1	Open	Open nephrectomy
Galt et al^4^	1995	10	One-stage	Open	Open nephrectomy
Konety et al^9^	1996	10	One-stage	Open	Open nephrectomy
Tsuji et al^10^	1999	1	One-stage	Open	Open nephrectomy
Hafez et al^3^	2000	27	One-stage: 11 Two-stage: 16	Open: 24 EVAR: 3	Open nephrectomy
Kouzai et al^13^**	2000	1	One-stage	Open	Open nephrectomy
Marrocco-Trischitta et al^14^	2001	1	One-stage	Open	Open nephrectomy
Toursarkissian et al^15^**	2001	1	One-stage	EVAR	Endovascular
Illuminati et al^16^	2004	1	One-stage	Open	Open nephrectomy
Veraldi et al^2^	2006	12	One-stage: 10 Two-stage: 2	Open	Open nephrectomy
Somani et al^17^	2009	1	One-stage	Open	Open nephrectomy
Pattaras & Milner^7^	2009	1	Two-stage	EVAR	Laparoscopic nephrectomy
Kira et al^5^	2012	1	Two-stage	EVAR	Laparoscopic nephrectomy
Sammut et al^18^	2012	1	One-stage	Open	Accessory artery ligation
Třeška et al^1^	2014	19	One-stage	Open	Open nephrectomy
Mozafar et al	2020	1	One-stage	Open	Open nephrectomy

AAA, abdominal aortic aneurysm; EVAR, endovascular aneurysm repair. *Partial or radical nephrectomy. **Horseshoe kidney.

In conclusion, single-stage surgical treatment of AAA and RCC is a safe and feasible approach. AAA is repaired by open technique followed by radical or partial nephrectomy. However, current data suggest endovascular management of AAA. In this approach, laparoscopic nephrectomy is conducted either in the same admission or a few weeks later. If a gastrointestinal malignancy accompanies RCC, the priority of cancer surgery and AAA surgery should be weighed. In the cases of metastatic cancer, EVAR is used followed by chemotherapy for cancer.

## Competing interests

None.

## Ethical approval

Ethical approval is not necessary for retrospective studied and case presentation in our institutional policies. However, informed consent has been obtained from the patient to publish this case.
